# Superimposing Status Epilepticus on Neuron Subset-Specific PTEN Haploinsufficient and Wild Type Mice Results in Long-term Changes in Behavior

**DOI:** 10.1038/srep36559

**Published:** 2016-11-07

**Authors:** Gregory D. Smith, Jessika White, Joaquin N. Lugo

**Affiliations:** 1 Institute of Biomedical Sciences, Baylor University, Waco, TX 76798, USA; 2 Department of Psychology and Neuroscience, Baylor University, Waco, TX 76798, USA

## Abstract

We evaluated the effects of superimposing seizures on a genetic mutation with known involvement in both Autism Spectrum Disorder and in epilepsy. Neuron-subset specific (NS)-Pten heterozygous (HT) and wildtype (WT) adult mice received either intraperitoneal injections of kainic acid (20 mg/kg) to induce status epilepticus or the vehicle (saline). Animals then received a battery of behavioral tasks in order to evaluate activity levels, anxiety, repetitive-stereotyped behavior, social behavior, learning and memory. In the open field task, we found that HT mice after seizures showed a significant increase in total activity and total distance in the surround region of the open field. In the elevated plus maze task, we found that HT mice after seizures displayed increased total distance and velocity as compared to HT mice that did not undergo seizures and WT controls. In the social chamber test, we found the HT mice after seizures displayed an impairment in social behavior. These findings demonstrate that superimposing seizures on a genetic mutation can result in long-term alterations in activity and social behavior in mice.

Individuals with a history of seizures have higher incidence rates for other comorbidities. There is increasing evidence for a strong relationship between epilepsy and autism, with the prevalence of epilepsy in children with autism spectrum disorder (ASD) ranging from 5–40%^1^,^2^,^3^. In one study with a cohort of almost 15,000 people, approximately 19% of those with epilepsy were also found to have ASD, while the controls were only found to have a 2% rate of ASD^4^. Although the variety of findings across studies may be due to seizure classification, ASD classification, study populations, and study methods, there is substantial support for the relationship between autism and epilepsy.

There are several mechanisms that may underlie the relationship between ASD and epilepsy. One possibility is that there is a common neurobiological vulnerability, such as brain development lesions or genetic susceptibilities that may lead to abnormal brain development, subsequently resulting in ASD and epilepsy. Another possible reason could be that the abnormal brain circuitry underlying ASD could lead to seizures. Overlap between these two mechanisms may also exist. Several genetic developmental disorders have been associated with an increased risk of developing epilepsy, including both fragile X syndrome (FXS)^3^,^5^ and tuberous sclerosis^3^,^6^.

Even though the genetic knockout models have provided insight into the neural mechanisms involved in autism and epilepsy, it has been difficult to differentiate the effects of seizures from the genetic condition. One genetic knockout model that may aid in parsing out these effects may be the *PTEN* deletion model. Phosphatase and tensin homolog (PTEN) acts as a negative repressor of the PI3K/AKT/mTOR intracellular signaling pathway^7^. Mouse models with a deletion of *PTEN* show learning and memory problems, autistic-like behavioral deficits^8^,^9^, and spontaneous seizures^10^. In neuron-subset specific Pten knockout (KO) mice, onset of seizure activity occurs at 4 weeks of age, with progressive seizure intensity as the animal develops^11^. The autistic-like behavioral deficits in neuron-subset specific Pten KO occur primarily at 6–8 weeks of age^12^. Given the staggered onset of seizure and behavioral phenotypes, it is not clear whether seizures are the cause of the behavioral deficits or are co-occurring with the behavioral deficits. Mice with germline haploinsufficiency in *PTEN* have social behavior deficits, but do not exhibit seizures^13^. Therefore, mice that are *PTEN* haploinsufficient may be useful in characterizing the effects of seizures on the magnitude of behavioral deficits.

Seizure induction has been shown to result in autistic-like behavioral deficits^14^,^15^. For this study, we attempted to characterize the impact of seizures on autistic-like behavioral phenotypes expressed by neuron subset-specific (NS)-Pten haploinsufficient mice. To establish typical ASD behaviors in mice, performance was observed in an array of behavioral tests known reliably to model the characteristic symptoms of ASD seen in human patients. Tests included the open field test and elevated plus maze to determine general behaviors, including activity and anxiety levels. Social interactions were examined using the three chamber social test. Hippocampal dependent learning and memory was tested using the Morris water maze and trace fear conditioning^16^,^17^. Lastly, the marble burying test was used to investigate repetitive behaviors^18^.

In addition, protein expression levels were examined using western blotting analyses. In animal models of Fragile X syndrome, tuberous sclerosis, and *PTEN* deletion, aberrant changes have been found in the PI3K/AKT/mTOR pathway^7^,^8^,^19^,^20^,^21^. Disruptions in the PI3K/AKT/mTOR pathway have shown to occur in approximately 14% of individuals diagnosed with ASD^22^,^23^. In one study with 114 patients, 8.3% of children with ASD had a mutation in *PTEN*^24^ and 12.2% of children demonstrated developmental delay/mental retardation^24^. All of the ASD patients with *PTEN* mutations had significant macrocephaly in their clinical study. Therefore, we examined several proteins with known involvement in the PI3K/AKT/mTOR signaling pathway, as well as others that are known to be aberrant in animal models of Fragile X syndrome, tuberous sclerosis, and *PTEN* deficiency.

## Results

### Seizure induction

A total of 59 mice survived seizure induction and were used in behavior testing. The WT-Saline group had 18 mice, HT-Saline group had 12 mice, WT-Seizure group had 15 mice, and HT-Seizure group had 14 mice. Of the 21 WT mice injected, 3 (14.3%) died, 3 were removed because they did not go into status epilepticus (14.3%), and the average onset to the initial seizure was 19.65 seconds. Of the 16 HT mice injected, 2 (12.5%) died with the average onset to the first seizure being 19.68 seconds. Only mice that entered into full status epilepticus were included in this study so that all mice fell into the same highest category of seizure severity.

#### Status epilepticus in the HT group resulted in hyperactivity in the open field test

**Figure 1:**
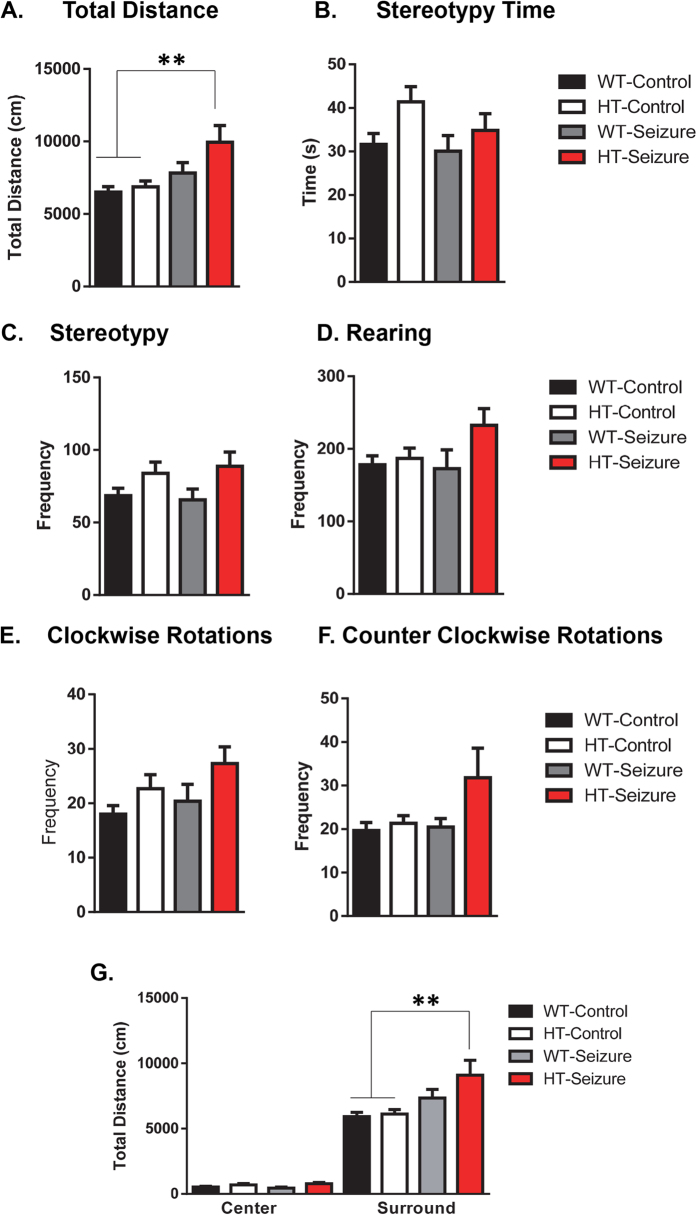
Status epilepticus in NS-Pten HT mice resulted in hyperactivity in the open field. The mice were placed in an open field test for thirty minutes and several measurements of locomotor behavior were taken. (**A**) Total distance, (**B**) time performing stereotypy behavior, (**C**) frequency of stereotypy events, (**D**) number of rearing events, (**E**) number of clockwise rotations, and (**F**) number of counterclockwise rotations. The data were then reanalyzed by center and surround regions of the open field apparatus. (**G**) Total distance in the center and surround region of the open field test. Data are shown as mean ± standard error of the mean. **P > 0.01. WT-control n = 17, HT-control n = 12, WT-Seizure n = 14, HT-Seizure n = 14.

There was a significant difference between groups in total distance K(3,53) = 13.68, p < 0.01 (Fig. 1A). Dunn’s post hoc tests revealed that the HT-seizure group mice traveled significantly greater distance when compared to the WT-control and HT-control groups. Nonparametric tests were used to account for unequal variances between groups. There was no difference in the number or total duration spent performing stereotypy actions F(3,53) = 2.3, p = 0.09 (Fig. 1B), which is a measure of self-grooming events, and no difference between groups in number of stereotypy events (Fig. 1C). However, using a Kruskal-Wallis test there was a significant effect of group in the number of rearing events K(3,53) = 8.1, p < 0.05 (Fig. 1D), however post hoc Dunn’s test did not show a significant difference between any of the groups. There was also a trending, but not significant effect in the number of clockwise rotations between groups K(4,57) = 7.31, p = 0.06 (Fig. 1E), and no significant differences in the number of counter clockwise rotations amongst the groups K(3,53) = 5.45, p = 0.14 (Fig. 1F).

When comparing total distance traveled in the surround vs. center of the open field test, there was a trending, but not statistically different group effect in the center area K(3,53) = 7.6, p = 0.056 (Fig. 1G Left Graph). There was a significant difference between groups in distance traveled in the surround area K(3,53) = 13.71, p < 0.01 (Fig. 1G Right Graph). Using Dunn’s multiple comparison test we found significant differences between the WT-Control and HT-Control, as compared to the HT-Seizure group. There was no significant difference in fecal boli production between groups F(3,55) = 0.57, p = 0.63, data not shown. For the open field analysis data there were 2 fewer mice reported, one from the WT-saline group and one from WT-seizure group, due to computer error following testing.

#### Status epilepticus in the HT group resulted in hyperactivity without changes in anxiety in the elevated plus maze test

**Figure 2:**
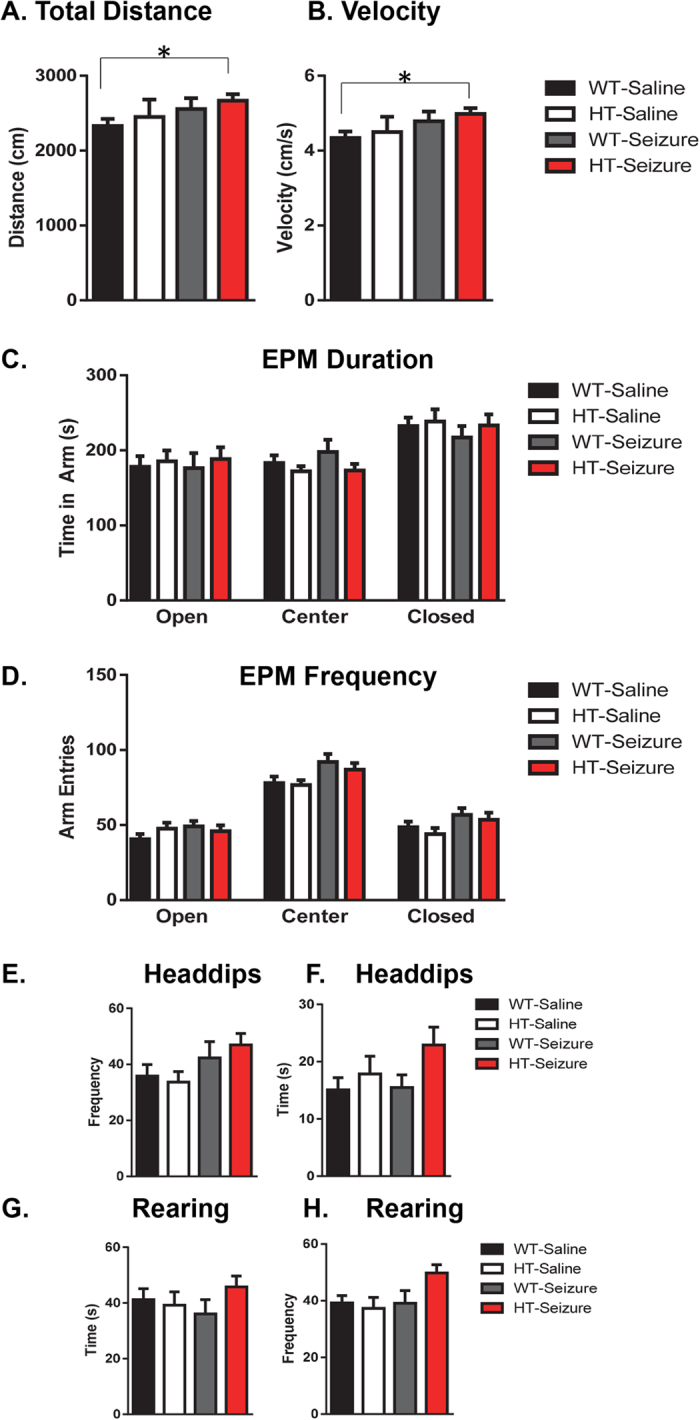
Status epilepticus in the NS-Pten HT mice resulted in hyperactivity without changes in anxiety in the elevated plus maze test. All groups of mice were placed in a 10 minute elevated plus maze test. We first examined (**A**) overall changes in total distance in the maze and (**B**) velocity. (**C**,**D**) Duration and frequency of visits in the open, center, and closed arms in the maze. Separate video scoring was conducted for the (**E**,**F**) frequency and duration of head dips and (**G**,**H**) frequency and duration of rearing behavior in the closed arms. Data are shown as mean ± standard error of the mean. *P > 0.05. WT-control n = 18, HT-control n = 12, WT-Seizure n = 15, HT-Seizure n = 14.

There was a significant effect in total distance traveled by mice in the elevated plus maze K(3,55) = 8.1, p < 0.05 (Fig. 2A). Dunn’s post hoc tests revealed a significant difference in distance between the WT-Control and HT-Seizure groups. Similar differences were observed in reference to velocity in the maze K(3,55) = 9.5, p < 0.05 (Fig. 2B). Dunn’s post hoc tests revealed a significant difference in velocity between the WT-Control and HT-Seizure groups.

For analysis of the open versus closed arms, the total time spent and number of entries into each type of arm was grouped together based on type. Center refers to the central section where all of the arms meet. There were no differences in time spent in the open arms F(3,55) = 0.12, p = 0.95; center F(3,55) = 1.01, p = 0.39; or in the closed F(3,55) = 0.41, p = 0.74 (Fig. 2C). Similar results were found in the frequency of visits to each arm for the open F(3,55) = 1.1, p = 0.37; center F(3,55) = 2.6, p = 0.06; and closed F(3,55) = 1.6, p = 0.19 (Fig. 2D). In reference to general exploratory behaviors during the elevated plus maze task, the number or head dips in the open arm and number of rearings in the closed arms were compared across the groups. There were no significant differences in head dipping frequency F(3,55) = 1.9, p = 0.13 (Fig. 2E) or duration F(3,55) = 1.6, p = 0.18 (Fig. 2F). There were no differences in the duration of rearing in the closed arms F(3,55) = 0.81, p = 0.5 (Fig. 2G). However, there was a trending, but not statistically significant effect in the number of rearing events in the closed arms F(3,55) = 2.5, p = 0.068 (Fig. 2H).

#### No differences between groups were found in repetitive behavior

**Figure 3:**
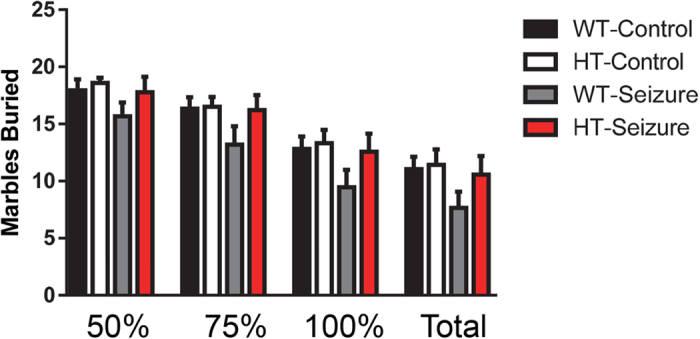
No difference in repetitive behavior was found between Pten wildtype and heterozygous mice after status epilepticus. There were no changes in the number of marbles buried in a 30 minute test. The number of marbles buried were examined at different levels: 50%, 75%, 100%, and completely buried. Data are shown as mean ± standard error of the mean. WT-control n = 18, HT-control n = 12, WT-Seizure n = 15, HT-Seizure n = 14.

There were no significant differences in the number of marbles buried at various depths between groups at any depth. There were no difference at the 50% F(3,55) = 1.3, p = 0.27; 75% F(3,55) = 1.6, p = 0.20; 100% F(3,55) = 1.6, p = 0.18; or completely buried marbles F(3,55) = 1.6, p = 0.20 (Fig. 3).

#### Status epilepticus in the HT group resulted in social behavior deficits

**Figure 4:**
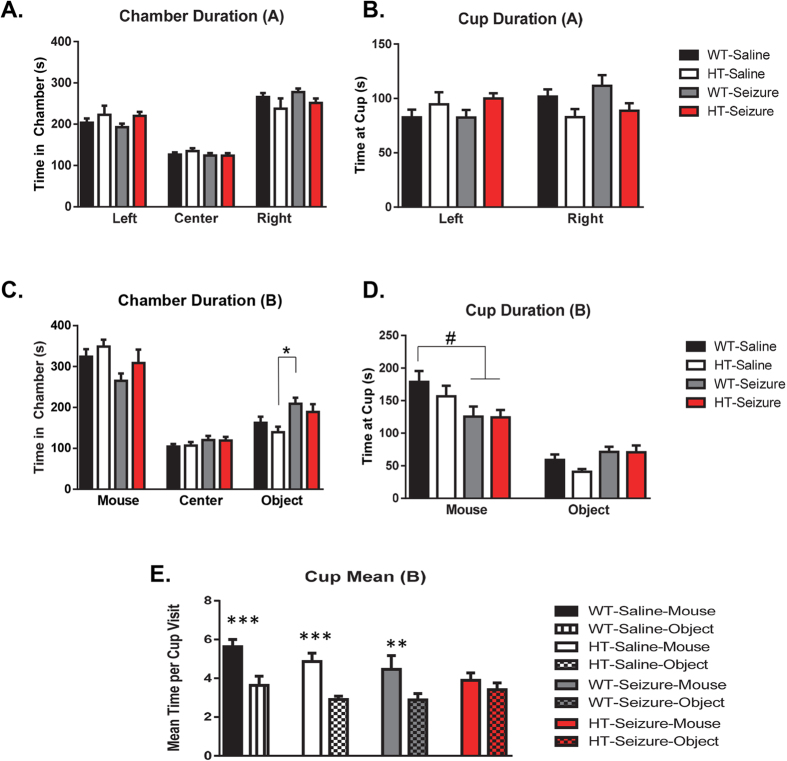
Status epilepticus in the NS-Pten HT mice resulted in social behavior deficits. (**A**) Time in the left, center, and right chamber in phase A. (**B**) Duration of interaction at the cups within the chamber in phase A. (**C**) Time in the chamber with the mouse, center, and chamber that housed the novel object. (**D**) Time spent with the cup that housed the mouse and object in the three chamber social behavior test. (**E**) Mean time interacting with the mouse compared to the novel object across groups. The values represent the mean ± SEM. ^#^*P* = 0.05; *P < 0.05; **P < 0.01; ***P < 0.001. WT-control n = 18, HT-control n = 12, WT-Seizure n = 15, HT-Seizure n = 14.

Phase A: For all phase A analyses, chambers and cups are referred to as left and right, where in phase B all sides are referred to as either mouse or object based on what was placed into the cup on that side. There were no significant differences between groups in duration of time in the chambers F(3,55) = 0.12, p = 0.85, but there was a significant difference across chambers F(2,110) = 91.4, p < 0.001 (Fig. 4A). There were no significant differences in duration of visit at the empty cups between groups F(3,55) = 0.4, p = 0.75 (Fig. 4B), as well as no differences in time spent at the cups F(1,55) = 1.2, p = 0.26.

Phase B Chamber: In phase B, there were no differences between the groups in the time spent between the chambers F(3,55) = 1.2, p = 0.317, but there was a significant difference across chambers F(2,110) = 103.0, p < 0.001 (Fig. 4C) and a significant interaction between groups across chambers F(6,110) = 2.6, p < 0.05. Separate One-Way ANOVA tests revealed a significant difference in the total time spent with the object F(3,55) = 3.4, p < 0.05, with Tukey post hoc tests revealing a significant difference between HT-Control and WT-Seizure groups. There was a trending, but not statistically significant effect in total time spent at the chamber that housed the mouse F(3,55) = 2.4, p = 0.081. Phase B Cups: When we analyzed the duration at the cups that housed the mice, there were no differences between groups in time spent at the cup F(3,55) = 2.1, p = 0.11 (Fig. 4D). There was a main effect of time spent at each cup F(1,55) = 74.6, p < 0.001, which suggests that the mice preferred to interact with the mouse compared to the object. There was a significant interaction between the groups across the cups F(3,55) = 3.6, p < 0.05. Separate one way ANOVA tests revealed a significant main effect of group in the time spent at the cup with the mouse F(3,55) = 3.2, p < 0.05. There were trending, but not statistically significant effects in Tukey post-hoc tests at the duration of time spent at the mouse cup with WT-Control compared to WT-Seizure and HT-Seizure (p = 0.06). We also measured the mean number of visits for the cup that housed the mouse and objects. There was no main effect of group F(3,55) = 1.5, p = 0.22 (Fig. 4E). However, there was a difference in mean time spent per cup F(1,55) = 59.3, p < 0.001. There was also a significant interaction of group x cup F(3,55) = 3.2, p < 0.05. There were no statistically significant differences between groups with a One-Way ANOVA. However, separate paired t-tests revealed a significant preference for the cup with the mouse over the object in all groups WT-Vehicle t(1,17) = 5.5, p < 0.001; HT-Vehicle t(1,11) = 4.8, p < 0.001; WT-Seizure t(1,14) = 3.6, p < 0.01; except the HT-Seizure t(1,13) = 1.5, p = 0.16. The paired t-test detected that the HT-Seizure group did not show a preference between the mouse and the object, revealing a social behavior deficit in the HT-Seizure group.

#### Status epilepticus in the HT group resulted in enhanced learning in the trace fear conditioning test

**Figure 5:**
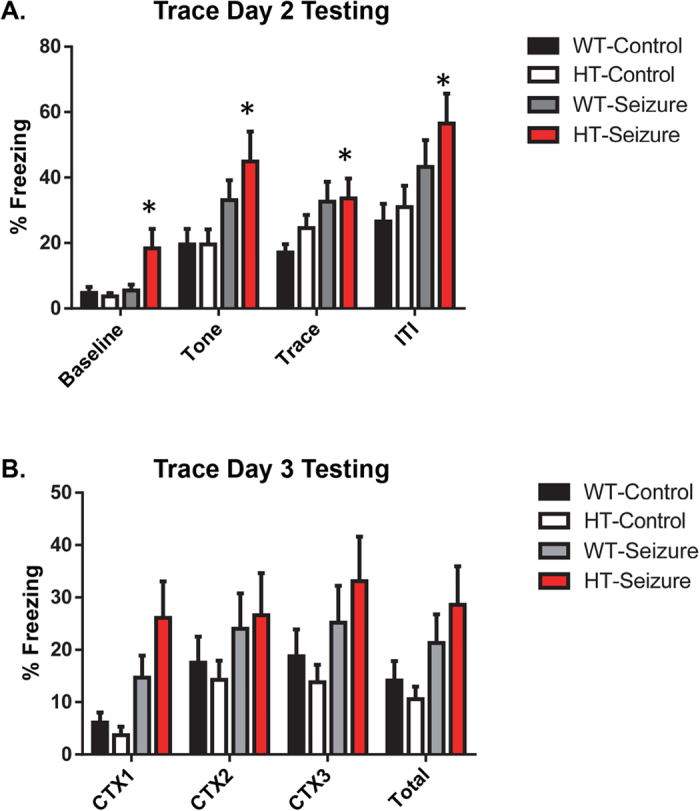
Status epilepticus in the HT group resulted in enhanced learning in the trace fear conditioning test. Mice were examined for differences in learning and memory through a trace fear conditioning test. (**A**) The HT mice with status epilepticus had more freezing across all trials. (**B**) No differences were observed in contextual conditioning a day later. Data are shown as mean ± standard error of the mean. *P < 0.05. WT-control n = 18, HT-control n = 12, WT-Seizure n = 15, HT-Seizure n = 14.

On the third day of testing, mice were placed in a novel context and a conditioned stimulus tone was presented 4 different times. The freezing levels of the mice were recorded during baseline, the conditioned stimulus, the trace period, and all inter-trial intervals. Results demonstrated a main effect of group F(3,55) = 4.0, p < 0.05 and a main effect of time F(3,165) = 54.9, p < 0.001 (Fig. 5A). Tukey post hoc tests revealed a significant difference between the WT-saline group compared to the HT-seizure group. The HT-seizure group displayed an increase in freezing across the entire testing period. Furthermore, there was a trending, but not statistically significant interaction between group and time F(9, 165) = 1.8, p = 0.078. On the fourth day, we tested the groups in the original context for three minutes. There was no main effect of group over the context test period F(3,55) = 2.3, p = 0.089, and no main effect of time F(6,110) = 0.75, p = 0.61 (Fig. 5B). There was also no interaction between group over time F(6,110) = 0.75, p = 0.61.

#### There were no spatial learning deficits after status epilepticus in the Morris water maze

**Figure 6:**
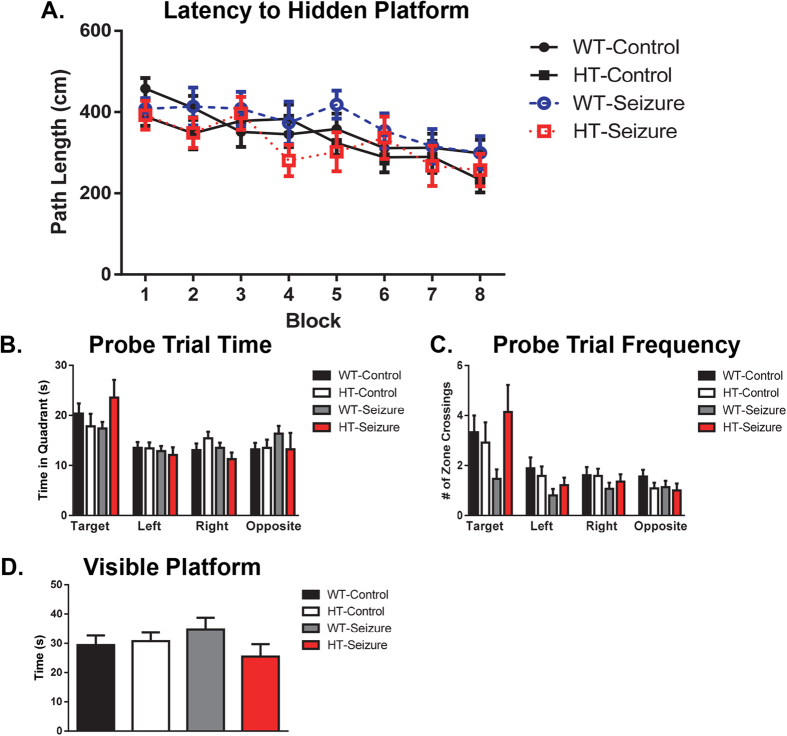
There were no spatial learning deficits after status epilepticus in the Morris water maze. The mice were trained over eight blocks to find the hidden platform. There were no impairments to find the hidden platform (**A**). After 8 blocks of learning were completed, the animals were tested for spatial memory retention by the probe trial. There were no differences in the amount of time in each quadrant (**B**) or in the number of crossings in each zone (**C**). There were no differences in visible platform testing (**D**). Data are shown as mean ± standard error of the mean. WT-control n = 18, HT-control n = 12, WT-Seizure n = 15, HT-Seizure n = 14.

Spatial Learning Acquisition: There were no differences between groups in path length F(3,55) = 0.67, p = 0.57, swim velocity F(3,55) = 0.7, p = 0.56 (data not shown), and time to find hidden platform (escape time) F(3,55) = 0.78, p = 0.51 (data not shown) across the 8 blocks (4 trials per block per mouse) of training for Morris water maze (Fig. 6A). All groups showed learning and improvement across training blocks. There was a significant main effect of time for path length, F(7,385) = 11.39, p < 0.0001, escape time, F(7,385) = 19.02, p < 0.0001, and swim velocity, F(7,385) = 14.84, p < 0.0001. There were no significant interactions between group and trial in any of the measures.

Probe Trial: The probe trial was run following the last training block on day 4. Following the last training block on day 4 the probe trial was performed. There was no difference in the duration of time in each quadrant between groups F(3,55) = 0.53, p = 0.66, but there was a significant difference across quadrants F(3,165) = 9.3, p < 0.001 (Fig. 6B). There was a trending, but not statistically significant effect of number of times in the zone where the hidden platform was located F(3,55) = 2.5, p = 0.073 (Fig. 6C). There was a main effect across zones F(3,165) = 14.7, p < 0.001. There were no significant interactions found between groups across the four quadrants.

Visible Platform: On the fifth day of testing the platform was made visible and two blocks of two trials were run. During the visible platform trials, no significant difference was found between groups in average escape time F(3,55) = 1.02, p = 0.39 (Fig. 6D).

#### No statistically significant changes were determined in the western blotting experiments

The mean and standard error of the means for the four groups are presented in Table 1. The table also includes the statistical information for comparisons of the four groups. There were no statistically significant differences found in examination of any of the proteins. The densitized values and representative western blots can be found in the supplemental figures.

## Discussion

The most consistent outcome we found in our study was hyperactivity of the Pten haploinsufficient mice that received status epilepticus (SE). The Pten haploinsufficient mice that received SE during adulthood had an increase in total activity in the open field test, an increase in total distance in the surround region of the open field, and an increase in total distance and velocity in the elevated plus maze task. Previous clinical and animal model studies provide support for the comorbidity of hyperactivity in epilepsy. Attention-deficit hyperactivity disorder (ADHD) has a prevalence rate of 7 to 9% in children and 2.5–4% in adults, along with evidence that individuals with epilepsy have 2–3 times higher rates of ADHD^25^. Animal models of epilepsy provide additional support for the comorbidity between epilepsy and hyperactivity. In one study, investigators induced SE in adult male Wistar rats with pilocarpine and found that half of the subjects displayed inattention and impulsivity^26^. For our experiments we only evaluated locomotor activity in the open field and elevated plus maze tests. Future studies could include a lateralized reaction-time task and other tests of impulsivity to examine changes in the subjects’ impulsivity and attention. One caveat to the open field test and elevated plus maze tests is that the tests occur only one time and are brief. Testing conditions such as day of testing, time of day, size of arena, length of test, and lighting conditions can all influence activity. Langford-Smith (2011) found that in their mouse model of a syndrome with a lysosomal storage disorder, a 1 hour testing trial 1 hour after the mice entered the light phase most reliably detected hyperactivity^27^. Future experiments could vary the open field testing paradigm and include a 24 h home-cage monitoring system to assess long-term activity measures of the NS-Pten HT mice with SE. These data from the open field and plus maze supports that the seizures in NS-Pten HT mice result in hyperactivity without changes in anxiety like behaviors.

The Pten haploinsufficient mice that received SE during adulthood also displayed a possible impairment of social behavior in the three chamber social test. Impairment in social behavior is one of the three core features of ASD. The WT-Seizure and HT-Seizure mice had a marginal decrease in the duration of time to investigate the cup that housed the mouse compared to the WT control group (Fig. 4D) during the second phase of the three chamber social behavior test. The WT control, HT control, and WT-Seizure mice all exhibited a significant preference for the mouse compared to the cup, while the HT-Seizure group did not (Fig. 4E). Other animal models of epilepsy have found that seizures induced during early development or induced during adulthood can result in social behavior deficits^14^,^15^,^28^. In addition, conditional Pten knockout mice have social behavior deficits^8^,^12^. However, a caveat in the conclusion of social behavior deficits in the HT-Seizure mice is that the mean time visit per cup is not a standard measure of sociability. Most studies use total duration of time in the chamber^29^ or percent time of investigation^30^,^31^. Therefore, future studies could include additional social behavior tests such as social partition to more fully examine the possible social behavior deficits. These data from the social chamber show that the HT-seizure group had an impairment in social behavior when compared to the control groups.

Contrary to previous studies, we did not observe deficits in social behavior for the Pten haploinsufficient mice that received a vehicle injection during adulthood. Existing studies using Pten germline haploinsufficient mice have found them to have social behavior deficits in the three chamber social behavior test^31^ as well as, decreased aggression and an increase in repetitive behavior^13^. One difference between the Pten haploinsufficient mice in these studies compared to the present study is that the Pten mouse they used had a germline mutation, while the mice used in our studies are instead a neuron-subset specific deletion of Pten using the Cre-loxP recombination system. The germline Pten haploinsufficient mice are known to have broad brain hypertrophy, while the NS-Pten HT mice have selective localized increases in brain overgrowth^32^. In addition, male mice with germline heterozygous deletion of Pten also have alterations in repetitive behavior and mood/anxiety^31^. For instance, the germline Pten HT mice have been found to bury more marbles in the marble burying test than the Pten WT mice. The Pten HT mice displayed a higher preference for the dark chamber over the light chamber in the light/dark test, which indicates a higher level of anxiety. The Pten HT mice were more immobile than WT in the tail suspension test and forced swim test. It may be that a constitutional heterozygous mutation in *PTEN* is necessary to influence social behavior. In addition, differences in genetic backgrounds may have influenced the behavioral outcomes of the germline heterozygous deletion of Pten. The Clipperton-Allen & Page, 2014 paper and others^30^,^33^,^34^ have used C57BL/6J background strain for the Pten knockout studies compared to the FVB strain used in the present study. Future studies may include crossing the NS-Pten deletion to the C57BL/6J background strain to investigate the influence of the genetic background.

One surprising result was the increase in freezing behavior displayed by the Pten HT-Seizure mice in the trace fear conditioning test. There are several possible interpretations for the enhanced freezing across all trials. One interpretation of the increase in freezing during tone, trace, and the ITI periods may be that Pten HT-Seizure mice demonstrated enhanced learning in the trace fear conditioning test. Even though we hypothesized that seizures in the Pten haploinsufficient mice would have learning deficits, previous studies have shown that seizures can instead lead to enhanced learning. Clinical research has found that school-aged children who experienced febrile seizures had significantly better learning, memory retrieval, consolidation, and delayed recognition compared to age-matched control children^35^,^36^. It has also been shown that adult mice that experienced febrile seizures on PD14 showed enhanced memory performance in contextual fear memory^37^. These mice have an increase in large mossy fiber terminals in the dentate gyrus of the hippocampus, which was suggested to correspond to the enhanced learning in these mice. A separate study found that febrile seizures induced in PD10 rat pups results in an increase in dendritic complexity of newborn dentate granule cells^38^. The most significant deletion of Pten in the NS-Pten mice is in the dentate gyrus. It is possible that seizures are enhancing some of the connections in the dentate gyrus in these mice. Future studies could further examine the alterations within the dentate gyrus of Pten mice. One interpretation of these data from the trace fear condition test is that the seizures resulted in enhanced learning in the NS-Pten.

Another interpretation of the enhanced freezing may be due to non-associative conditioning or pseudoconditioning. In the standard trace fear paradigm there is a time interval between the tone and shock. Through repeated pairings the animal will learn to predict the shock by the presentation of the tone. During the memory test the animal will increase its freezing behavior when presented with the tone. However, the animal may have a generalized increase in freezing regardless of the stimulus. In one study, the investigators found an increase in heart rate when the CS and US were paired and not when they were unpaired^39^. They found that a delay of 60 seconds or more between the CS and US eliminated the subsequent increase in heart rate in the fear conditioning test. Future experiments could include a group that receives unpaired presentations of the US and CS or an interval of 60 seconds or greater between the CS and US to reduce the impact of non-associative conditioning such as sensitization in fear conditioning. In addition, future studies could examine whether the NS-Pten HT mice with SE have higher levels of sensitization.

We performed western blotting studies on a number of proteins in the hippocampus of WT and HT mice with and without seizures. We found no differences between groups in the protein levels we measured in the hippocampus. We hypothesized that we would find hyperactivation in the PI3K/AKT/mTOR signaling pathway, given that this effect has been shown in other animal models after seizures^40^,^41^,^42^. However, an important consideration is that we did not observe spontaneous seizures in our mice. One study found that using pentylenetetrazole, which induces acute seizures and does not result in later spontaneous seizures, only results in temporary hyperactivation of the mTOR pathway^40^. The persistent long-term increase in mTOR activation occurs when there is confirmation of epileptogenesis in the animal model^41^. In our study we induced SE for 30 minutes because of high mortality rates when we extended the period to over 1 hour (preliminary results). The increase in susceptibility may be unique to the FVB strain that we used for our Pten WT and HT mice. Future studies may use intrahippocampal injections of kainate, as this method results in epileptogenesis and overall decreases the mortality rate in mice^42^. Additional future studies could look at different time points following seizure induction to see if any temporary hyperactivation of the mTOR pathway are seen. Another consideration would be to correlate seizure activity during the initiation of status epilepticus to changes in mTOR activation.

The western blots for the current study confirm our previous results with the NS-Pten WT and HT mice^9^. In our previous study we examined the mTOR pathway and FMRP in the hippocampus of WT, HT, and KO mice. We also examined trace fear conditioning and delay fear conditioning in NS-Pten WT, HT, and KO mice. We found no significant differences in protein levels for total and phosphorylated AKT, S6, FMRP, or in total levels of S6K1 for NS-Pten WT and HT mice. We also found no learning and memory deficits in WT and HT mice in trace fear conditioning or delay fear conditioning. The alterations we found in learning and memory and in the PI3K/AKT/mTOR pathway were in the NS-Pten KO mice only^9^,^12^.

Our study provides support to existing evidence that seizures superimposed on a genetic condition can result in behavioral comorbidities. We found that the Pten haploinsufficient mice with SE were hyperactive and showed some degree of impairment in social behavior. Several studies have used a “two-hit” model to examine how seizure induction during early development can increase susceptibility to seizures and behavioral comorbidities later in life^43^,^44^. Only recently has the “two-hit” model included a genetic deletion along with an induction of seizures. In one study, investigators administered electrical kindling in the Tg2576 mouse of model of Alzheimer’s disease^45^. They found that the Tg2576 mice are more susceptible to electrically evoked seizures. Even though the authors did not examine behavioral comorbidities, they provide additional evidence that a “two-hit” exposure to seizures can result in additional neurological deficits. Future studies could further examine the impact of seizures superimposed on genetic mutations in mice that develop spontaneous seizures or demonstrate susceptibility to seizures. Taking all of these data and looking at the behavior difference in the HT-seizure group support that the heterozygosity of the mice is the first hit and then the seizure induction is the second hit in our two-hit model.

## Materials and Methods

### Animals

For this study male neuron subset-specific Pten (NS-Pten) conditional mice, previously described as GFAP-Cre; Pten^loxP/loxP^^10^,^32^ were utilized. This conditional KO has been shown to be localized in the hippocampus primarily in the granule cells of the dentate gyrus^30^. Most Cre expression in the dentate gyrus granule cells occurs by postnatal day 5. The mice were on a FVB-based mixed background strain that had been bred for more than 10 generations. We bred NS-Pten^loxP/+^ heterozygote parents to produce NS-Pten^+/+^ wild type (WT) and Pten^loxP+^ heterozygous (HT). Mice were all generated and group housed at Baylor University, on a 14 hour light 10 hour dark diurnal cycle, at an ambient temperature of 22 °C. All mice were given *ad libitum* access to both food and water. All mice began testing at age postnatal day (PD) 60 and testing for each set of mice began at the same time of day (approximately 11 a.m.) for all testing. Only male mice were used in this study based on earlier studies using NS-Pten WT and KO “that” did not show differences in male and female mice of this strain^11^,^14^,^46^,^47^.

### Behavioral observation of kainate-induced seizures

Seizure induction began with mice on PD 60. Seizures were induced using kainic acid (Catalog # 0222; Tocris, Bristol, UK) that was suspended in 0.9% saline and administered by intraperitoneal (ip) injection (20 mg/kg). Controls were administered equivalent doses of 0.9% physiological saline vehicle. The presence of behavioral status epilepticus was determined using previously described methods^48^. At approximately 1 hour following either saline or kainic acid injections all mice received an IP injection of (20 mg/kg) of pentobarbital to terminate seizure activity.

### Behavior Testing

Following seizure induction or saline injection all mice were given a 1 week recovery period before behavioral testing was conducted over a 4 week period. Week 1 included open field testing followed by 1–2 rest days then elevated plus maze testing. Week 2 began with marble burying followed by 1–2 rest days then social chamber testing. Trace fear conditioning was tested over 4 days during week 3 and Morris water maze was performed over 5 days during week 4. Following behavior testing all mice were sacrificed and brains were removed and dissected to isolate the hippocampus. The hippocampi were then stored at −80 °C until the tissue could be processed and used in western blotting analyses.

### Locomotor activity: Open Field test

The open field test was used to determine locomotor activity, exploratory behaviors, and anxiety using previously described methods^12^. All activity was recorded using an optical recording system controlled by Fusion for the 30 minute test (Omnitech Electronics, Inc., Columbus, OH).

### Anxiety: Elevated Plus Maze Test

The elevated plus maze was used to test anxiety levels in the mice. The maze consists of an elevated plus platform with two enclosed arms and two open arms. Total distance and time spent in each of the arms was recorded for the 10 minute trial using previously described methods^12^. Mouse activity and movements were tracked using the video tracking software Noldus (Ethovision, Netherlands). Simultaneous video recording was done using video capturing software Dazzle video creator plus HD (Corel, Canada).

### Repetitive behaviors: Marble Burying

Marble burying was used to test repetitive behaviors using previously described methods^12^. Repetitive behaviors are one of the three core behavioral features of ASD. Test cages were set up with clean bedding (approximately 2 cm) and a 4 × 5 array of 20 marbles. All marbles used were black 20 mm opaque glass marbles. The mice were placed into the test cage and left for 30 minutes. The number of marbles that were buried at various depths (50%, 75%, 100%, and completely buried) were counted. The more marbles buried indicated repetitive behavior tendencies^18^.

### Social Behavior: Three-chamber test

Social behaviors were tested using a three chamber apparatus using previously described methods^12^. Social behavior is one of the three core behavioral features of ASD. The test was divided into two phases. In the first phase (phase A) a mouse was placed in a three chamber apparatus, where they can explore the testing arena and all three chambers and both cups for a total of 10 minutes. Each of the side chambers of the arena contained an empty wire mesh cup. Following phase A the mouse was returned to the central chamber and the doors to the adjacent chambers were blocked. A novel or stranger mouse (same-sex, weight and age) was placed in one cup and a novel small object roughly the same size and color as a mouse, was placed in the other cup. The location of the stranger mouse was alternated across mice to reduce preference bias for chamber location. All stranger mice were male WT mice from the C57BL/6J strain (similar age and size). The stranger mice were habituated to the cup by spending 1 hour a day in the cup on both of the two days prior to testing. During phase B, the mice were again allowed to freely explore the testing arena for 10 minutes. The time spent in each chamber and interacting with each of the cups was recorded. The amount time spent interacting with the novel mouse is a measure of sociability^29^. The video was captured using Dazzle video creator plus HD (Corel, Canada), for scoring at a later time.

### Hippocampal-Dependent Memory: Trace Fear Conditioning

We used trace fear conditioning to examine hippocampal dependent learning and memory, as learning disabilities and developmental delay/mental retardation are often seen with ASD. The freezing behaviors of the mice were recorded using Freeze Frame monitoring system (Coulbourn, Ohio, USA) and the methods have been used previously^17^,^49^. On the first day, mice were allowed to habituate to the chamber. On the second day, a tone was paired with an aversive stimulus (0.5 mA scrambled shock) after a 20 second trace period. On test day 3 the mice were returned to the test chamber, this time under novel contextual cues (see below for description). After a 180 second baseline period the mice were presented with a 20 second CS followed by an 80 second ITI. There were a total of 4 tone presentations during the 580 second test phase. On test day 4 the mice were placed in the test chamber under the original contextual setup and their freezing behavior in the chamber was recorded for a total of 180 seconds with no stimuli.

The testing chamber consisted of two clear acrylic sides and two metal sides with a metal bar floor that can receive a scrambled electrical current. The chamber was 26 cm × 22 cm × 18 cm and was placed in a sound attenuated outer chamber to control for background noise and light. On novel contextual testing days the floor of the chamber was replaced by a white foam pad under a clear acrylic square, and an additional clear acrylic wall was added diagonally across the chamber giving the mice access to half the chamber in a triangular form. An additional house light and fan was turned on in the testing chamber to alter the background light and noise levels. The apparatus was cleaned with 70% ethanol instead of 30% isopropanol, and a tray of vanilla extract was placed in the chamber to alter the smell. The testing room lights were dimmed and the bedding in the transfer cages was replaced with shredded paper to alter the context of transferring the mice from the holding room to the testing chamber. Two identical chambers were set up, in order to allow for testing of two different mice simultaneously.

### Spatial Memory: Morris Water Maze

It has been shown that Morris water maze can be used to determine hippocampal dependent spatial learning^16^. Spatial memory was tested because of the learning disabilities and developmental delay/mental retardation that are often seen with ASD. The procedure was previously described and consists of training the mice to find a hidden platform in the water maze with 2 blocks of training per day for 4 days^50^. Each block has four trials, each continuing until the mouse made it onto the platform, or 1 min of swimming had passed. Following each trial, the mouse was held on the platform for approximately 10 seconds before starting the next trial. The latency to escape, swim distance, and swim speeds were recorded. The last block, on day 4, was followed by a probe trial where the hidden platform was removed and the mice were placed in the maze for 1 minute. For the probe trial, the time spent in each quadrant and number of platform crossings (area where the platform was previously located) was recorded. On day 5 a visible platform test was given to make sure the vision and swimming ability of the mice was not a factor in longer escape latencies. During the visible platform training a total of 2 blocks consisting of 2 trials each was administered. Longer escape latency to the hidden platform over the eight trials and equal time spent in the target zone vs. non-target zones and/or fewer platform crossings during the probe trial demonstrates a deficit in spatial learning. Testing was recorded using the video tracking software Noldus (Ethovision, Netherlands).

### Western Blotting

One week after the completion of behavior testing, the mice were euthanized and the hippocampus was rapidly dissected out of the brain, rinsed in 1X PBS on ice, and then placed in dry ice prior to storing at −80 °C. Tissue was processed by previously described methods and consists of first homogenizing both hippocampi in homogenizing buffer (0.32 M sucrose, 1 mM EDTA, 5 mM Hepes) containing protease inhibitor cocktail (Sigma, St. Louis, MO)^51^. We then produced crude synaptosomes that were run through 8–12% SDS-PAGE gels and then were transferred to Hybond-P polyvinyl difluoride membranes (GE Helthcare, Piscataway, NJ). To produce the crude synaptosomes we first placed the tissue in a 2 ml Kontes^®^ tissue grinder glass tube. We then homogenized the tissue with a glass pestle for 20–25 strokes per tissue sample. We kept a 150 μL aliquot of the homogenized tissue solution for the total homogenate samples and added 50 μL of 4X sample buffer with the loading dye. We then centrifuged the remaining amount at 800 G for 10 minutes at 4 °C. We transferred the supernatant to another tube then spun the supernatant at 7200 G for 15 minutes at 4 °C. We removed the supernatant and resuspended the pellet with homogenizing buffer for use in western blotting as a crude synaptosome. We also added a 4X sample buffer solution that contained the loading dye. Blocking solution was prepared [5% non-fat milk in 1X Tris Saline (50 mM Tris-HCl, pH 7.4, 150 mM NaCl) with 0.1% Tween (1X TTBS) and 1 mM Na3VO4], and the membranes were incubated in the blocking solution for 1 hour at room temperature. Following blocking, membranes were incubated over night at 4 °C with primary antibodies in blocking solution (see Table 1 for antibody specifics). Following primary antibody incubation, membranes were then washed 3 times for 5 minutes each in TTBS, then incubated for 1 hour at room temperature with horseradish peroxidase labeled secondary antibodies in the milk solution at a concentration of 1:20000. After another three 5 minute washes in TTBS, membranes were incubated for 5 minutes at room temperature in GE ECL Prime, (GE Healthcare, Piscataway, NJ). The chemiluminescence immunoreactive bands were imaged using a digital western blot imaging system (ProteinSimple, Santa Clara, CA). Western blots were run for the primary antibody of concern. We then confirmed that a nonspecific band from the original protein of interest was not at the same location for the loading control by examining the western blot of the longest exposure image. We then reran the blots with the antibody for the loading control (actin or mortalin). See Table 1 for details on antibodies used.

### Statistical Analyses

Single measurement comparisons were analyzed using one-way ANOVAs or similar nonparametric tests (Kruskal-Wallis), when necessary to account for unequal variances. For repeated measure tests, data were analyzed using two-way repeated-measures ANOVA. All statistical and data analyses were conducting using Prism 6 (GraphPad Software, La Jolla, CA) or SPSS 21 (IBM, Armonk, NY).

## Ethics Statement

All procedures concerning animals were approved by the Baylor University Institutional Animal Care and Use Committee and followed in accordance to the approved Institutional and NIH guidelines.

## Additional Information

**How to cite this article**: Smith, G. D. *et al*. Superimposing Status Epilepticus on Neuron Subset-Specific PTEN Haploinsufficient and Wild Type Mice Results in Long-term Changes in Behavior. *Sci. Rep.* **6**, 36559; doi: 10.1038/srep36559 (2016).

**Publisher’s note:** Springer Nature remains neutral with regard to jurisdictional claims in published maps and institutional affiliations.

## Supplementary Material

Supplementary Information

## Figures and Tables

**Table 1:** Western blotting data analysis, summary of results (Values are mean ± SEM).

Protein	WT-Saline	HT-Saline	WT-Seizure	HT-Seizure	Sig./Change	Prep Used
AKT	100.0 ± 1.5	108.5 ± 9.0	98.8 ± 1.7	112.4 ± 7.8	—	Total
phospho-AKT (s473)	100.0 ± 15.9	101.5 ± 19.3	105.2 ± 14.2	92.2 ± 11.3	—	Total
% Total phospho-AKT (s473)	98.9 ± 14.8	97.5 ± 20.5	106.7 ± 14.3	82.3 ± 9.0	—	Total
S6	100.0 ± 5.7	96.5 ± 4.6	96.0 ± 4.6	86.2 ± 14.3	—	Total
phospho-S6 (s235/236)	100.0 ± 15.7	92.0 ± 19.2	105.4 ± 20.0	78.9 ± 16.5	—	Total
phospho-S6 (s240/244)	100.0 ± 2.6	104.7 ± 9.2	107.0 ± 9.6	96.7 ± 3.8	—	Total
% Total phospho- S6 (s235/236)	99.30 ± 14.0	93.5 ± 18.7	116.2 ± 21.3	120.8 ± 26.7	—	Total
% Total phospho- S6 (s240/244)	103.4 ± 8.7	110.7 ± 11.2	125.4 ± 18.2	173.5 ± 44.6	—	Total
p70 S6 kinase	100.0 ± 2.9	116.3 ± 33.3	91.0 ± 12.4	87.8 ± 14.9	—	Total
Ankyrin	100.0 ± 7.7	95.2 ± 9.1	86.3 ± 9.7	89.2 ± 8.2	—	Synap.
FMPR	100.0 ± 14.8	119.6 ± 18.6	110.5 ± 18.3	74.8 ± 16.3	—	Synap.
phospho-FMRP (s499)	100.0 ± 22.1	109.6 ± 19.2	110.0 ± 38.9	80.2 ± 15.7	—	Synap.
% Total phospho-FMRP (s499)	94.5 ± 14.3	106.3 ± 27.2	91.3 ± 19.5	297.5 ± 182	—	Synap.
GluR1	100.0 ± 21.2	74.0 ± 18.2	91.1 ± 21.1	105.2 ± 41.3	—	Synap.
HCN1	100.0 ± 19.0	86.3 ± 13.7	84.5 ± 21.5	64.5 ± 19.0	—	Synap.
Kv4.2	100.0 ± 11.5	111.6 ± 13.6	92.2 ± 7.6	82.9 ± 10.4	—	Synap.
mGluR1/5	100.0 ± 24.1	169.0 ± 54.9	93.5 ± 23.3	123.8 ± 26.6	—	Synap.
Pan Shank	100.0 ± 10.1	112.1 ± 7.31	120.6 ± 15.2	93.2 ± 5.8	—	Synap.
Pan SAPAP 110	100.0 ± 12.7	119.7 ± 18.5	101.7 ± 18.1	81.3 ± 11.8	—	Synap.
Pan SAPAP 120	100.0 ± 22.8	144.1 ± 42.8	200.9 ± 98.7	115.3 ± 35.6	—	Synap.
PSD 95	100.0 ± 5.2	99.96 ± 3.6	110.4 ± 6.9	100.2 ± 3.1	—	Synap.
Sample size	n = 8	n = 8	n = 10	n = 10		
