# Supplementary material for: Superimposing Status Epilepticus on Neuron Subset-Specific PTEN Haploinsufficient and Wild Type Mice Results in Long-term Changes in Behavior
Source: Sci Rep. 2016 Nov 7;6:36559. doi: 10.1038/srep36559 (PMC5098193; doi:10.1038/srep36559)
Supplement: Supplementary Information [file srep36559-s1.pdf]

**Supplemental figures for:**

# Superimposing Status Epilepticus on Neuron Subset-Specific PTEN Haploinsufficient and Wild Type Mice Results in Long-term Changes in Behavior

Gregory D. Smith[1], Jessika White[2], and Joaquin N. Lugo*[1,2]

[1]Institute of Biomedical Sciences, Baylor University, Waco, TX 76798, USA

[2]Department of Psychology and Neuroscience, Baylor University, Waco, TX 76798, USA

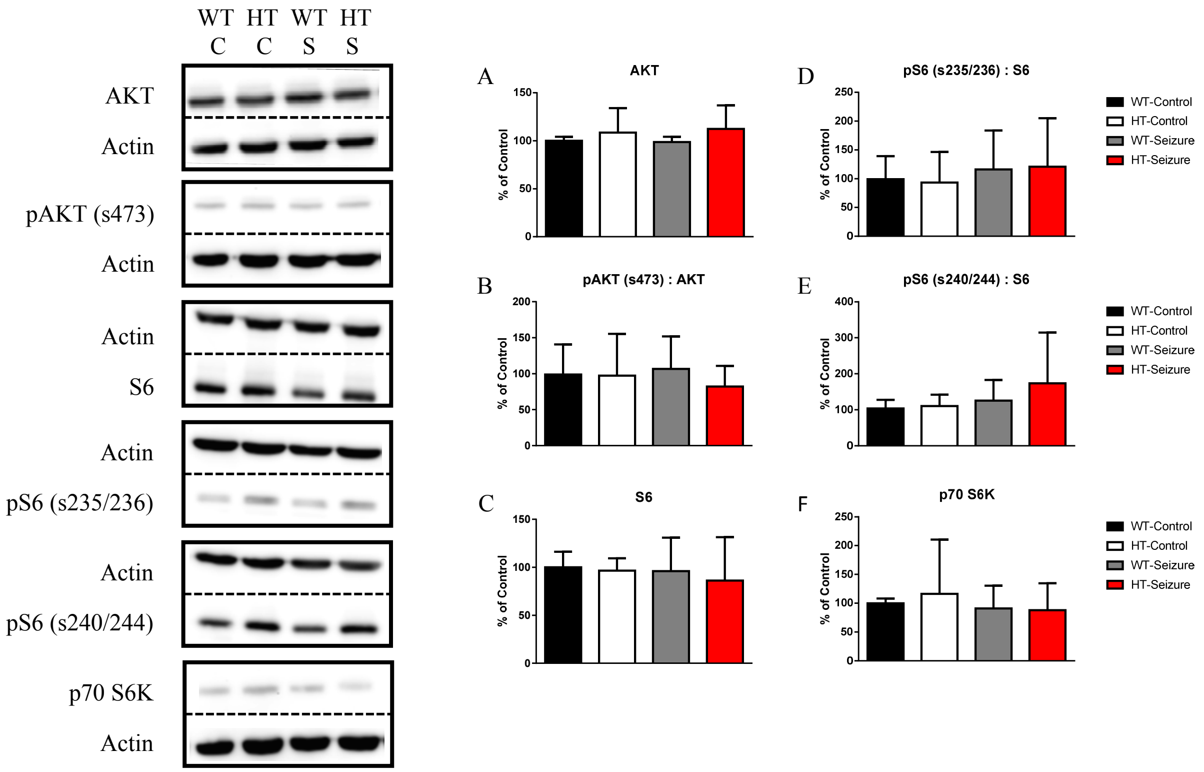


**Figure S1. Western blotting data for hippocampal total homogenate samples.** Left: Image of the western blot. Right: Relative changes in protein concentration normalized to the loading constant and expressed as a percentage of the control group. All phosphorylated proteins are expressed as a percentage of phosphorylated protein to total protein concentration. **(A)** AKT. **(B)** pAKT (s473). **(C)** S6. **(D)** pS6 (s235/236). **(E)** pS6 (s240/244). **(F)** p70 S6K. WT-control n=8, HT-control n=8, WT-Seizure n=10, HT-Seizure n=10.

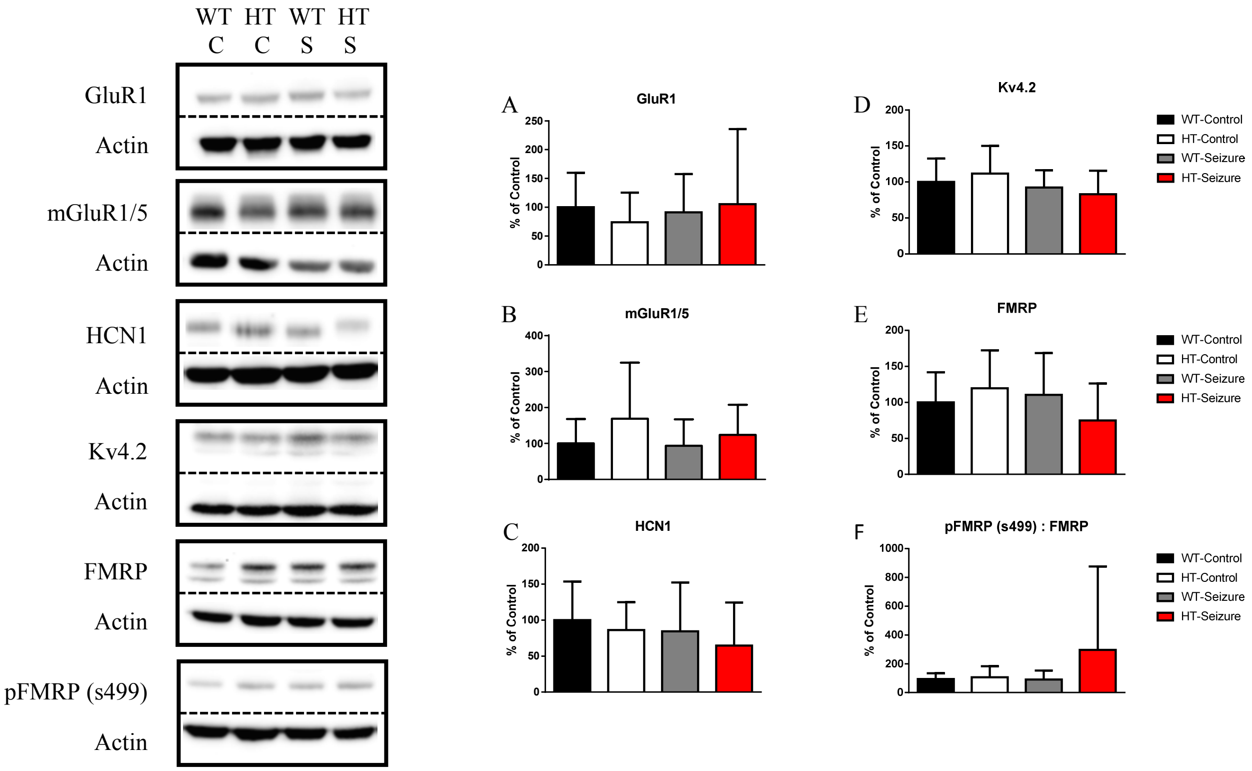


**Figure S2. Western blotting data for hippocampal synaptosome samples.** Left: Image of the western blot. Right: Relative changes in protein concentration normalized to the loading constant and expressed as a percentage of the control group. All phosphorylated proteins are expressed as a percentage of phosphorylated protein to total protein concentration. **(A)** GluR1. **(B)** mGluR1/5. **(C)** HCN1. **(D)** Kv4.2. **(E)** FMRP. **(F)** pFMRP (s499). WT-control n=8, HT-control n=8, WT-Seizure n=10, HT-Seizure n=10.

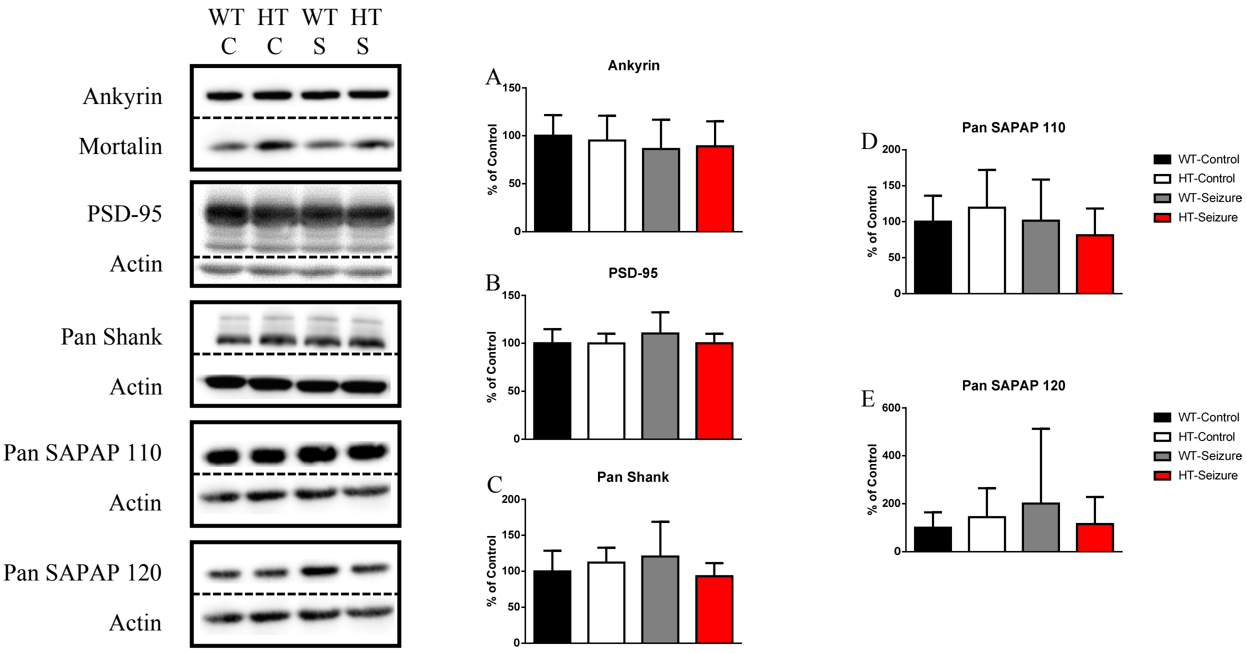


**Figure S3. Western blotting data for hippocampal synaptosome samples.** Left: Image of the western blot. Right: Relative changes in protein concentration normalized to the loading constant and expressed as a percentage of the control group. **(A)** Ankyrin. **(B)** PSD-95. **(C)** Pan Shank. **(D)** Pan SAPAP 110 kDa. **(E)** Pan SAPAP 120. WT-control n=8, HT-control n=8, WT-Seizure n=10, HT-Seizure n=10.
